# Supplementary material for: Surveillance, Isolation, and Genetic Characterization of Bat Herpesviruses in Zambia
Source: Viruses. 2023 Jun 13;15(6):1369. doi: 10.3390/v15061369 (PMC10303238; doi:10.3390/v15061369)
Supplement: Supplementary file 1 [file viruses-15-01369-s001.zip › viruses-2438686-supplementary.pdf]

Table S1 Primers used in this study

Primer	Sequence (5'-3')	Purpuse	Reference
DFASA-F	GTGTTCGACTTYGCNAGYYTNTAYCC	Screening (semi-nested) PCR	[23]
VYGA-nested-F	ACGTGCAACGCGGTGTAYGGNKTNACNGG		
GDTD1B-R	CGGCATGCGACAAACACGGAGTCNGTRTCNCCRTA		
Strain82-GAP1-F	CCAGAGCTGAGTTTGCCAAC	Nanopore-sequencing	In this study
Strain82-GAP1-R	AAAGTGCCACTAGACCCAAC		
Strain82-GAP2-F	TTGGACCACTATGGTGATGG		
Strain82-GAP2-R	TCTAACCTGACCAGGCAAGC		
Strain82-GAP3-F	TCTTAGGTGTGACTCAAACC		
Strain82-GAP3-R	GCCAGTAACTAGGAAGCACC		

Table S2 Reference sequences used in this study

Subfamily	Virus	Accession No.
Alfaherpesvirinae	Anatid alphaherpesvirus 1	NC_013036
	Ateline alphaherpesvirus 1	NC_034446
	Beluga whale alphaherpesvirus 1	NC_055166
	Bovine alphaherpesvirus 2	AF181249
	Bovine alphaherpesvirus 2	NC_043048
	Bovine alphaherpesvirus 5	NC_005261.3
	Bovine herpesvirus type 1.1	NC_063268
	Bubaline alphaherpesvirus 1	NC_043054
	Canid alphaherpesvirus 1	MW353126
	Caprine alphaherpesvirus 1	NC_043055
	Cercopithecine alphaherpesvirus 2	NC_006560
	Cercopithecine alphaherpesvirus 9	NC_002686.2
	Cervid alphaherpesvirus 1	NC_043056
	Cervid alphaherpesvirus 3	NC_055561
	Chelonid alphaherpesvirus 5	HQ878327.2
	Columbid alphaherpesvirus 1	NC_034266
	Equid alphaherpesvirus 1	NC_001491.2
	Equid alphaherpesvirus 3	NC_024771
	Equid alphaherpesvirus 4	NC_001844
	Equid alphaherpesvirus 8	KF434388
	Equid alphaherpesvirus 9	NC_011644
	Equid herpesvirus 6	MT012704
	Falconid herpesvirus 1	NC_024450
	Felid alphaherpesvirus 1	KR381779
	Fruit bat alphaherpesvirus 1	NC_024306
	Gallid alphaherpesvirus 1	NC_006623

	Gallid alphaherpesvirus 2	KU744558
	Gallid alphaherpesvirus 3	NC_002577
	Human alphaherpesvirus 1	ON783216
	Human alphaherpesvirus 1 strain 17	NC_001806.2
	Human alphaherpesvirus 2	MH697447
	Human alphaherpesvirus 2 strain HG52	NC_001798.2
	Human alphaherpesvirus 3	NC_001348
	Leporid alphaherpesvirus 4	NC_029311
	Macacine alphaherpesvirus 1	NC_004812
	Macropodid alphaherpesvirus 1	NC_029132
	Macropodid alphaherpesvirus 2	MT900475
	Meleagrid alphaherpesvirus 1	NC_002641
	Papiine alphaherpesvirus 2	NC_007653
	Phocid alphaherpesvirus 1	MH509440.2
	Psittacid alphaherpesvirus 1	NC_005264
	Saimiriine alphaherpesvirus 1	NC_01456
	Spheniscid alphaherpesvirus 1	LT608136
	Suid alphaherpesvirus 1	NC_006151
	Testudinid alphaherpesvirus 3	NC_027916.2
	Walrus alphaherpesvirus 1	LC532169
Betaherpesvirinae	Aotine betaherpesvirus 1	NC_016447
	Caviid betaherpesvirus 2	NC_020231
	Cebine betaherpesvirus 1	NC_038260
	Cercopithecine betaherpesvirus 5	NC_012783.2
	Cynomolgus cytomegalovirus	NC_033176
	Cynomolgus macaque cytomegalovirus strain Ottawa	NC_016154
	Elephant endotheliotropic herpesvirus 4	NC_028379
	Elephant endotheliotropic herpesvirus 5	NC_024696
	Elephantid betaherpesvirus 1	NC_020474.2
	Human betaherpesvirus 5	NC_006273.2
	Human betaherpesvirus 6A	NC_001664.4
	Human betaherpesvirus 6B	NC_000898
	Human betaherpesvirus 7	NC_001716.2
	Macaca nemestrina herpesvirus 7	NC_030200
	Macacine betaherpesvirus 3	NC_006150
	Murid betaherpesvirus 1	NC_004065
	Murid betaherpesvirus 8	NC_019559.2
	Murine roseolovirus	NC_033620
	Panine betaherpesvirus 2	NC_003521
	Papio ursinus cytomegalovirus	NC_027016
	Rat cytomegalovirus strain Maastricht	NC_002512.2
	Saimiriine betaherpesvirus 4	NC_016448

	Suid betaherpesvirus 2	NC_022233
	Tupaiid betaherpesvirus 1	NC_002794
Gammaherpesvirinae	Alcelaphine gammaherpesvirus 1	NC_002531
	Alcelaphine gammaherpesvirus 2	NC_024382
	Ateline gammaherpesvirus 3	NC_001987
	Bovine gammaherpesvirus 4	NC_002665
	Bovine gammaherpesvirus 6	NC_024303
	Callitrichine gammaherpesvirus 3	NC_004367
	Callitrichine gammaherpesvirus 3	NC_004367
	Colobine gammaherpesvirus 1 Hannover strain	MH932584
	Common bottlenose dolphin gammaherpesvirus 1 strain Sarasota	NC_035117
	Cricetid gammaherpesvirus 2	NC_015049
	Eptesicus fuscus gammaherpesvirus	NC_040615
	Equid gammaherpesvirus 2 strain 275	HQ247750
	Equid gammaherpesvirus 2 strain 2B	HQ247756
	Equid gammaherpesvirus 2 strain 37	HQ247753
	Equid gammaherpesvirus 2 strain 86/67	NC_001650.2
	Equid gammaherpesvirus 2 strain ATCC	HQ247755
	Equid gammaherpesvirus 2 strain G9/92	KM924294
	Equid gammaherpesvirus 5 strain 2-141/67	NC_026421
	Equid gammaherpesvirus 5 strain 281	GQ325593
	Equid gammaherpesvirus 5 strain Archey	KC715730
	Equid gammaherpesvirus 5 strain Chantilly Lake	KC715732
	Equid gammaherpesvirus 5 strain SJ	KC715731
	Felis catus gammaherpesvirus 1	NC_028099
	Harp seal herpesvirus	NC_055139
	Human gammaherpesvirus 4 AIL14 strain	MH837517
	Human gammaherpesvirus 4 strain B95-8	NC_007605
	Human gammaherpesvirus 8 strain GK18	NC_009333
	Human gammaherpesvirus 8 VG-1 strain	GU233088
	Human herpesvirus 4 type 2 strain AG876	NC_009334
	Lymphocryptovirus Macaca/pfe-lcl-E3	NC_055142
	Macaca fuscata rhadinovirus	NC_007016
	Macaca mulatta rhadinovirus NEPRC 159-05 strain	GU233144
	Macaca nemestrina rhadinovirus 2	NC_055141
	Macacine gammaherpesvirus 4	NC_006146
	Macacine gammaherpesvirus 5	NC_003401
	Murid gammaherpesvirus 4	NC_001826.2
	Mustelid gammaherpesvirus 1	NC_038266
	Myotis gammaherpesvirus 8	NC_029255
	Myotis ricketti herpesvirus 1	JN692429
	Myotis ricketti herpesvirus 2	JN692430

Ovine gammaherpesvirus 2	NC_007646
Panine gammaherpesvirus 1	NC_038859
Phascolarctid gammaherpesvirus 1	NC_055555
Pongine gammaherpesvirus 2	NC_038860
Porcine lymphotropic herpesvirus 1	NC_038264
Porcine lymphotropic herpesvirus 2	NC_038265
Porcine lymphotropic herpesvirus 3	NC_055234
Retroperitoneal fibromatosis-associated herpesvirus	NC_055135
Rhinolophus gammaherpesvirus 1	NC_040539
Saimiriine gammaherpesvirus 2	NC_00135
Tursiops truncatus gammaherpesvirus V1977 MIA0501 strain	KX494870
Vombatid gammaherpesvirus 1	NC_055554
Wood mouse herpesvirus	NC_055233

Table S3 Highest amino acid identities for partial DPOL genes detected by screening

Subfamily	Virus	% identity	Closest virus	Acession No.
Betaherpesvirinae	*Macronycteris vittatus*/Zambia/64/2018	64.4	*Miniopterus natalensis*/South Africa/UP5701/2015	AYC44580
Betaherpesvirinae	*Macronycteris vittatus*/Zambia/50/2018	55.0	*Miniopterus natalensis*/South Africa/UP6250/2016	AYC44579
Betaherpesvirinae	*Macronycteris vittatus*/Zambia/20/2018	58.3	*Miniopterus schreibersii*/Spain/psc-13/2002or2004or2007	AMY98771
Betaherpesvirinae	*Macronycteris vittatus*/Zambia/36/2018	58.3	*Miniopterus schreibersii*/Spain/psc-13/2002or2004or2007	AMY98771
Betaherpesvirinae	*Macronycteris vittatus*/Zambia/41/2018	60.0	*Miniopterus schreibersii*/Spain/psc-13/2002or2004or2007	AMY98771
Betaherpesvirinae	*Macronycteris vittatus*/Zambia/46/2018	58.3	*Miniopterus schreibersii*/Spain/psc-13/2002or2004or2007	AMY98771
Betaherpesvirinae	*Macronycteris vittatus*/Zambia/101/2018	60.0	*Miniopterus schreibersii*/Spain/psc-13/2002or2004or2007	AMY98771
Betaherpesvirinae	*Macronycteris vittatus*/Zambia/104/2018	60.0	*Miniopterus schreibersii*/Spain/psc-13/2002or2004or2007	AMY98771
Betaherpesvirinae	*Rousettus aegyptiacus*/Zambia/10/2018	100	*Rousettus aegyptiacus*/South Africa/UP5647/2015	AYC44561
Betaherpesvirinae	*Rousettus aegyptiacus*/Zambia/11/2018	96.6	*Rousettus aegyptiacus*/South Africa/UP6435/2016	AYC44560
Betaherpesvirinae	*Macronycteris vittatus*/Zambia/37/2018	45.6	Phacochoerus africanus cytomegalovirus 1	AAP42116
Betaherpesvirinae	*Macronycteris vittatus*/Zambia/49/2018	45.6	Phacochoerus africanus cytomegalovirus 1	AAP42116
Betaherpesvirinae	*Macronycteris vittatus*/Zambia/106/2018	45.6	Phacochoerus africanus cytomegalovirus 1	AAP42116
Betaherpesvirinae	*Rousettus aegyptiacus*/Zambia/3/2018	60.5	*Plecotus austriacus*/Spain/psc-23/2004or2007	AMY98781
Betaherpesvirinae	*Rousettus aegyptiacus*/Zambia/6/2018	60.5	*Plecotus austriacus*/Spain/psc-23/2004or2007	AMY98781
Betaherpesvirinae	*Macronycteris vittatus*/Zambia/114/2018	60.5	*Plecotus austriacus*/Spain/psc-23/2004or2007	AMY98781
Betaherpesvirinae	*Macronycteris vittatus*/Zambia/126/2018	60.5	*Pteropus giganteus*/Bangladesh/PgHV-10/2006-2010	AGW27603
Betaherpesvirinae	*Macronycteris vittatus*/Zambia/84/2018	75.0	*Rhinolophus ferrumequinum*/Spain/psc-24/2004or2007or2008	AMY98782
Gammaherpesvirinae	*Macronycteris vittatus*/Zambia/22/2018	86.0	*Rhinolophus blythi*/China/RB-13YF84/2013	ALH21090
Gammaherpesvirinae	*Macronycteris vittatus*/Zambia/30/2018	84.2	*Rhinolophus blythi*/China/RB-13YF84/2013	ALH21090
Gammaherpesvirinae	*Macronycteris vittatus*/Zambia/38/2018	87.7	*Rhinolophus blythi*/China/RB-13YF84/2013	ALH21090
Gammaherpesvirinae	*Macronycteris vittatus*/Zambia/39/2018	82.5	*Hipposideros pomona*/China/HP-11HN110/2011	ALH21054
Gammaherpesvirinae	*Macronycteris vittatus*/Zambia/80/2018	86.0	*Rhinolophus blythi*/China/RB-13YF84/2013	ALH21090
Gammaherpesvirinae	*Macronycteris vittatus*/Zambia/82/2018	84.2	*Hipposideros pomona*/China/HP-11HN110/2011	ALH21054
Gammaherpesvirinae	*Hipposideros caffer*/Zambia/112/2018	87.7	*Hipposideros pomona*/China/HP-11HN110/2011	ALH21054
Gammaherpesvirinae	*Macronycteris vittatus*/Zambia/21/2018	77.2	*Hipposideros pomona*/China/HP-11HN110/2011	ALH21054

Gammaherpesvirinae	*Macronycteris vittatus*/Zambia/23/2018	75.4	*Hipposideros pomona*/China/HP-11HN110/2011	ALH21054
Gammaherpesvirinae	*Macronycteris vittatus*/Zambia/28/2018	77.2	*Hipposideros pomona*/China/HP-11HN110/2011	ALH21054
Gammaherpesvirinae	*Macronycteris vittatus*/Zambia/29/2018	77.2	*Hipposideros pomona*/China/HP-11HN110/2011	ALH21054
Gammaherpesvirinae	*Macronycteris vittatus*/Zambia/42/2018	75.4	*Hipposideros pomona*/China/HP-11HN110/2011	ALH21054
Gammaherpesvirinae	*Macronycteris vittatus*/Zambia/45/2018	75.4	*Hipposideros pomona*/China/HP-11HN110/2011	ALH21054
Gammaherpesvirinae	*Macronycteris vittatus*/Zambia/55/2018	77.2	*Hipposideros pomona*/China/HP-11HN110/2011	ALH21054
Gammaherpesvirinae	*Macronycteris vittatus*/Zambia/92/2018	77.2	*Hipposideros pomona*/China/HP-11HN110/2011	ALH21054
Gammaherpesvirinae	*Macronycteris vittatus*/Zambia/94/2018	77.2	*Hipposideros pomona*/China/HP-11HN110/2011	ALH21054
Gammaherpesvirinae	*Macronycteris vittatus*/Zambia/96/2018	77.2	*Hipposideros pomona*/China/HP-11HN110/2011	ALH21054
Gammaherpesvirinae	*Macronycteris vittatus*/Zambia/25/2018	68.4	*Miniopterus schreibersi*/China/MS-11HN95/2011	ALH21055
Gammaherpesvirinae	*Rousettus aegyptiacus*/Zambia/4/2018	100	*Rousettus aegyptiacus*/Hungary/XXX/2007	ACY82599
Gammaherpesvirinae	*Rousettus aegyptiacus*/Zambia/7/2018	82.5	*Acerodon celebensis*/Indonesia/IFB13-28/2013	BBA93921
Gammaherpesvirinae	*Rousettus aegyptiacus*/Zambia/15/2018	100	*Rousettus aegyptiacus*/Hungary/XXX/2007	ACY82599

Individual herpesviruses detected from bats are named as follows: bat species of origin/country of identification/common name/year of collection.

Table S4 Identity comparison of each viral protein among Macronycteris gammaherpesvirus 1 strains and rhinolophus gammaherpesvirus 1

Protein	Amino acid sequence identity (%) between		Product or predicted function
	MaGHV1 and RGHV1	Strains 80 and 82	
BM1	-	N/A	homolog of CASP8 and FADD-like apoptosis regulator
BM2	-	N/A	hypothetical protein
BM3	41	60.1	homolog of EHV2 E3 membrane protein E3
BM4	42.1	94.4	homolog of E3 ubiquitin-protein ligase and RGHV1 ORF4 MIR-like membrane protein
BM5	62.2	94	homolog of EHV2 E4 apoptosis regulator BALF1
ORF6	71.1	99.3	single-stranded DNA binding protein
ORF7	60.7	97.9	DNA packaging terminase subunit 2
ORF8	72	92.9	glycoprotein B
ORF9	78.4	98.2	DNA polymerase catalytic subunit
BM6	34.5	100	bcl-2-like protein
BM7	58.8	100	homolog of EHV2 E6 membrane protein BILF1
ORF10	34.3	98	homolog of EHV2 ORF10 protein G10
ORF11	53.9	96.8	homolog of EHV2 ORF11 virion protein G11
BM8	39	95.5	homolog of bovine herpesvirus 6 Bov8 putative major envelope glycoprotein, RGHV1 ORF18 glycoprotein
ORF17	49	99	capsid maturation protease
ORF17.5	42.7	98.2	capsid scaffold protein
ORF18	55.9	100	homolog of EHV2 ORF18 protein UL79
ORF19	61.2	98.7	homolog of EHV2 ORF19 DNA packaging tegument protein UL25
ORF20	71.4	99.1	homolog of EHV2 ORF20 nuclear protein UL24
ORF21	58.7	98.6	thymidine kinase
ORF22	61.2	96	glycoprotein H
ORF23	53.4	96.9	homolog of EHV2 ORF23 tegument protein UL88
ORF24	58.8	99.7	homolog of EHV2 ORF24 protein UL87
ORF25	78.1	99.9	major capsid protein
ORF26	74	99	capsid triplex subunit 2
ORF27	29.5	91	homolog of EHV2 ORF27 envelope glycoprotein 48
ORF28	36.8	95.1	homolog of EHV2 ORF28 envelope glycoprotein 150
ORF29a	70.2	97.4	DNA packaging terminase subunit 1
ORF29b	76.3	98.8	DNA packaging terminase subunit 1
ORF30	53.6	95.2	homolog of EHV2 ORF30 protein UL91
ORF31	61.3	98.6	homolog of EHV2 ORF31 protein UL92

ORF32	51.8	96	homolog of EHV2 ORF32 DNA packaging tegument protein UL17
ORF33	47.1	95.1	homolog of EHV2 ORF33 tegument protein UL16
ORF34	63.8	98.1	homolog of EHV2 ORF34 protein UL95
ORF35	63.5	99.3	homolog of EHV2 ORF35 tegument protein UL14
ORF36	60.5	96.7	tegument serine/threonine protein kinase
ORF37	68	99.3	deoxyribonuclease
ORF38	38.6	90.4	myristylated tegument protein
ORF39	63.6	98.9	glycoprotein M
ORF40a	43.64	95.8	helicase-primase subunit
ORF40b	46.86	100	helicase-primase subunit
ORF42	62.8	100	homolog of EHV2 ORF42 tegument protein UL7
ORF43	80.2	100	capsid portal protein
ORF44	78.6	100	helicase-primase helicase subunit
ORF45	47.1	89.5	homolog of EHV2 ORF45 tegument protein G45
ORF46	75.8	97.2	uracil-DNA glycosylase
ORF47	48.6	78.2	glycoprotein L
ORF48	43.2	97	homolog of EHV2 ORF48 tegument protein G48
ORF49	42.1	97.9	homolog of EHV2 ORF49 tegument protein G49
ORF50	37.3	93.3	protein Rta
BM9	43.6	93.6	hypothetical protein, homolog of RGHV1 ORF53
BM10	-	98.5	hypothetical protein
BM11	50	83	homolog of EHV2 E7A envelope glycoprotein 42
BM12	42.9	52.6	hypothetical protein, homolog of RGHV1 ORF57
ORF52	53.2	94.7	homolog of EHV2 ORF52 virion protein G52
ORF53	71.9	97.7	glycoprotein N
ORF54	59	96.5	dUTPase
ORF55	78.7	99.5	homolog of EHV2 ORF55 tegument protein UL51
ORF56	67.6	98.8	helicase-primase primase subunit
ORF57	61.7	95.5	multifunctional expression regulator
ORF58	58.2	95	homolog of EHV2 ORF58 envelope protein UL43
ORF59	48.6	96.5	DNA polymerase processivity subunit
ORF60	80.7	98.3	ribonucleotide reductase subunit 2
ORF61	63.4	96.6	ribonucleotide reductase subunit 1
ORF62	62.7	98.1	capsid triplex subunit 1
ORF63	50.2	96.7	homolog of EHV2 ORF63 tegument protein UL37
ORF64	48.2	96.5	large tegument protein
ORF65	42.9	93.9	small capsid protein
ORF66	50.6	96.5	homolog of EHV2 ORF66 protein UL49

ORF67	74.8	98.9	nuclear egress membrane protein
ORF67A	65.9	96.6	homolog of EHV2 ORF67A DNA packaging protein UL33
ORF68	59.9	98.6	envelope glycoprotein, DNA packaging protein UL32
ORF69	67.7	100	nuclear egress lamina protein
ORF70	71	99.6	thymidylate synthase
BM13	-	88.2	hypothetical protein
ORF73	30.9	N/A	nuclear antigen LANA-1
ORF74	47.5	100	homolog of EHV2 ORF74 membrane protein G74
ORF75	54.2	98.5	homolog of EHV2 ORF75 tegument protein G75
BM14	-	99	hypothetical protein
BM15	-	98.4	hypothetical protein
BM16	-	100	hypothetical protein

-: No significant similarity was found by BLAST analyses.

Abbreviations: N/A, not analyzed; MaGHV1, Macronycteris gammaherpesvirus 1; RGHV1; rhinolophus gammaherpesvirus 1; EHV2, equine gammaherpesvirus 2.

Table S5 Predicted protein coding regions in the genome of Macronycteris gammaherpesvirus 1 strain 80

Gene	Contig	Location (nucleotides)	Strand	Size (no. of amino acids)	Product or predicted function
BM1	None	None	None	None	homolog of CASP8 and FADD-like apoptosis regulator
BM2	None	None	None	None	hypothetical protein
BM3	1	926-1,999	+	357	homolog of EHV2 E3 membrane protein E3
BM4	1	2,910-3,074	-	54	homolog of E3 ubiquitin-protein ligase and RGHV1 ORF4 MIR-like membrane protein
BM5	1	3,442-3,999	+	185	homolog of EHV2 E4 apoptosis regulator BALF1
ORF6	1	4,201-7,599	+	1,132	single-stranded DNA binding protein
ORF7	1	7,669-9,717	+	682	DNA packaging terminase subunit 2
ORF8	1	9,733-12,282	+	849	glycoprotein B
ORF9	1	12,553-15,558	+	1,001	DNA polymerase catalytic subunit
BM6	1	15,593-16,105	-	170	bcl-2-like protein
BM7	1	16,303-17,406	+	367	homolog of EHV2 E6 membrane protein BILF1
ORF10	1	17,450-18,847	+	465	homolog of EHV2 ORF10 protein G10
ORF11	1	18,907-20,142	+	411	homolog of EHV2 ORF11 virion protein G11

BM8	2	364-2,019	-	551	homolog of bovine herpesvirus 6 Bov8 putative major envelope glycoprotein, RGHV1 ORF18 glycoprotein
ORF17	2	2,159-3,739	-	526	capsid maturation protease
ORF17.5	2	2,159-3,010	-	284	capsid scaffold protein
ORF18	2	3,732-4,508	+	258	homolog of EHV2 ORF18 protein UL79
ORF19	2	4,505-6,127	-	540	homolog of EHV2 ORF19 DNA packaging tegument protein UL25
ORF20	2	5,991-6,695	-	234	homolog of EHV2 ORF20 nuclear protein UL24
ORF21	2	6,694-8,310	+	538	thymidine kinase
ORF22	2	8,310-10,535	+	741	glycoprotein H
ORF23	2	10,532-11,725	-	397	homolog of EHV2 ORF23 tegument protein UL88
ORF24	2	11,780-13,972	-	730	homolog of EHV2 ORF24 protein UL87
ORF25	2	13,977-18,110	+	1,377	major capsid protein
ORF26	2	18,130-19,032	+	300	capsid triplex subunit 2
ORF27	2	19,033-19,473	+	146	homolog of EHV2 ORF27 envelope glycoprotein 48
ORF28	2	19,538-19,789	+	83	homolog of EHV2 ORF28 envelope glycoprotein 150
ORF29a	2	24,132-25,292	-	386	DNA packaging terminase subunit 1
ORF29b	2	19,879-20,922	-	347	DNA packaging terminase subunit 1
ORF30	2	21,057-21,311	+	84	homolog of EHV2 ORF30 protein UL91
ORF31	2	21,224-21,907	+	227	homolog of EHV2 ORF31 protein UL92
ORF32	2	21,853-23,208	+	451	homolog of EHV2 ORF32 DNA packaging tegument protein UL17
ORF33	2	23,201-24,367	+	388	homolog of EHV2 ORF33 tegument protein UL16
ORF34	2	25,291-26,259	+	322	homolog of EHV2 ORF34 protein UL95
ORF35	2	26,246-26,716	+	156	homolog of EHV2 ORF35 tegument protein UL14
ORF36	2	26,613-27,914	+	433	tegument serine/threonine protein kinase
ORF37	2	27,917-29,371	+	484	deoxyribonuclease
ORF38	2	29,326-29,517	+	63	myristylated tegument protein
ORF39	2	29,584-30,717	-	377	glycoprotein M
ORF40a	2	30,828-32,261	+	477	helicase-primase subunit
ORF40b	2	32,289-32,819	+	176	helicase-primase subunit
ORF42	2	32,814-33,629	-	271	homolog of EHV2 ORF42 tegument protein UL7
ORF43	2	33,616-35,319	-	567	capsid portal protein
ORF44	2	35,285-37,651	+	788	helicase-primase helicase subunit
ORF45	2	37,705-38,607	-	300	homolog of EHV2 ORF45 tegument protein G45
ORF46	2	38,609-39,367	-	252	uracil-DNA glycosylase
ORF47	2	39,330-39,788	-	152	glycoprotein L
ORF48	2	39,886-41,319	-	477	homolog of EHV2 ORF48 tegument protein G48

ORF49	2	41,680-42,564	-	294	homolog of EHV2 ORF49 tegument protein G49
ORF50	2	42,709-44,280	+	523	protein Rta
BM9	2	44,414-44,719	+	101	hypothetical protein, homolog of RGHV1 ORF53
BM10	2	44,978-45,385	+	135	hypothetical protein
BM11	2	45,498-46,175	+	225	homolog of EHV2 E7A envelope glycoprotein 42
BM12	2	46,185-47,003	+	272	hypothetical protein, homolog of RGHV1 ORF57
ORF52	2	47,017-47,421	-	134	homolog of EHV2 ORF52 virion protein G52
ORF53	2	47,476-47,736	-	86	glycoprotein N
ORF54	2	47,826-48,695	+	289	dUTPase
ORF55	2	48,764-49,417	-	217	homolog of EHV2 ORF55 tegument protein UL51
ORF56	2	49,390-51,957	+	855	helicase-primase primase subunit
ORF57	2	52,162-53,589	+	475	multifunctional expression regulator
ORF58	2	54,452-55,489	-	345	homolog of EHV2 ORF58 envelope protein UL43
ORF59	2	55,498-56,727	-	409	DNA polymerase processivity subunit
ORF60	2	56,889-57,806	-	305	ribonucleotide reductase subunit 2
ORF61	2	57,834-60,149	-	771	ribonucleotide reductase subunit 1
ORF62	2	60,173-61,174	-	333	capsid triplex subunit 1
ORF63	2	61,185-63,968	+	927	homolog of EHV2 ORF63 tegument protein UL37
ORF64	2	63,973-70,950	+	2,325	large tegument protein
ORF65	2	70,964-71,464	-	166	small capsid protein
ORF66	2	71,475-72,785	-	436	homolog of EHV2 ORF66 protein UL49
ORF67	2	72,692-73,531	-	279	nuclear egress membrane protein
ORF67A	2	73,594-73,863	-	89	homolog of EHV2 ORF67A DNA packaging protein UL33
ORF68	2	73,966-75,348	+	460	envelope glycoprotein, DNA packaging protein UL32
ORF69	2	75,350-76,213	+	287	nuclear egress lamina protein
ORF70	2	76,279-77,157	-	292	thymidylate synthase
BM13	2	77,382-77,693	+	103	hypothetical protein
ORF73	None	None	None	Non	nuclear antigen LANA-1
ORF74	3	488-1,462	+	324	homolog of EHV2 ORF74 membrane protein G74
ORF75	3	1,625-5,698	-	1,357	homolog of EHV2 ORF75 tegument protein G75
BM14	3	5,732-6,061	+	109	hypothetical protein
BM15	3	6,387-6,980	+	197	hypothetical protein
BM16	3	7,578-8,081	+	167	hypothetical protein
